# Nanomaterials: Synthesis and Applications in Theranostics

**DOI:** 10.3390/nano11123228

**Published:** 2021-11-28

**Authors:** Gokul Paramasivam, Vishnu Vardhan Palem, Thanigaivel Sundaram, Vickram Sundaram, Somasundaram Chandra Kishore, Stefano Bellucci

**Affiliations:** 1 Department of Biotechnology, Saveetha School of Engineering, Saveetha Institute of Medical & Technical Sciences (SIMATS), Saveetha Nagar, Thandalam, Chennai 602 105, Tamil Nadu, India; gokul.parama@gmail.com (G.P.); vishnuvardhanp.sse@saveetha.com (V.V.P.); vickramas.sse@saveetha.com (V.S.); schandrakishore30@gmail.com (S.C.K.); 2 INFN—Laboratori Nazionali di Frascati, 00044 Frascati, Italy

**Keywords:** nanomaterials, top-down approach, bottom-up approach, quantum dots, CNT, graphene, nanocubes, therapy, diagnosis, theranostics

## Abstract

Nanomaterials are endowed with unique features and essential properties suitable for employing in the field of nanomedicine. The nanomaterials can be classified as 0D, 1D, 2D, and 3D based on their dimensions. The nanomaterials can be malleable and ductile and they can be drawn into wires and sheets. Examples of nanomaterials are quantum dots (0D), nanorods, nanowires (1D), nanosheets (2D), and nanocubes (3D). These nanomaterials can be synthesized using top-down and bottom-up approaches. The achievements of 0D and 1D nanomaterials are used to detect trace heavy metal (e.g., Pb^2+^) and have higher sensitivity with the order of five as compared to conventional sensors. The achievements of 2D and 3D nanomaterials are used as diagnostic and therapeutic agents with multifunctional ability in imaging systems such as PET, SPECT, etc. These imaging modalities can be used to track the drug in living tissues. This review comprises the state-of-the-art of the different dimensions of the nanomaterials employed in theranostics. The nanomaterials with different dimensions have unique physicochemical properties that can be utilized for therapy and diagnosis. The multifunctional ability of the nanomaterials can have a distinct advantage that is used in the field of theranostics. Different dimensions of the nanomaterials would have more scope in the field of nanomedicine.

## 1. Introduction

Theranostics is a term that evolved from the fusion of two words, therapeutics and diagnostics, which involves the usage of radioactive drugs to diagnose and to deliver therapy for treating any disease. In essence, theranostics is essential in treating human epidermal growth factor receptor 2 (HER 2) in breast cancer treatment with anti-HER 2 receptor antibodies [1], radioiodine therapy for differentiated thyroid cancers [2].

Therapeutic radiopharmaceuticals such as ^186^Re and ^188^Re—hydroxyethylidene diphosphonic acid (HEDP) and ^153^Sm-ethylenediamine tetramethylene phosphonic acid (EDTMP) are used for the treatment of metastatic bone lesions [3]. Most of these theranostic agents were assessed in the situation of evidence-based medications and compared to the effects of other conventional treatments. Theranostics is also essentially involved in the treatment of any disease on the molecular level for identification, diagnosis, and treatment [4].

In this context, nanotechnology is an emerging field of research in biomedical applications. Nanoparticles are part of the technology as a molecular probe for detecting and curing diseases. The nanoparticles are very small and are 10^−9^ meters in size. It has unique physical and chemical properties when compared to bulk materials [5]. The broad classification of nanoparticle synthesis is used of top-down and bottom-up techniques. The top-down technique involves physical participation approaches such as mechanical machining, physical vapor deposition (PVD), lithography, and pyrolysis through thermal evaporation pyrolysis [6]. Bottom-up methods consist of chemical and biological approaches. Sol-gel, chemical vapor deposition (CVD), chemical co-precipitation, micro-emulsions, hydrothermal method, sonochemical, and microwave methods are involved in the bottom-up chemical approaches [7]. Furthermore, other methods of synthesizing nanoparticles are through plant extracts, enzymes, agricultural waste, microorganisms, and actinomycetes [8]. Synthesized nanoparticles are allowed to characterize the property of the nanomaterials. There are a variety of nanoparticles that have been synthesized and applied to biomedical fields. Some of these include quantum dots, magnetic nanoparticles, optically active nanoparticles, carbon nanoparticles, emulsions, micelles, liposomes, microcapsules, microspheres, and thin films [9]. Each nanoparticle has its own unique characteristic and is beneficial for sensing, detecting, and diagnosing any disease. Exposure of nanoparticles in the biofluid shows aggregation, which tends to lose its properties. Additionally, nanoparticles with multifunctional features are necessary in biomedical applications. The scientific field is expected to produce multiple insights from the same nanomaterials [10].

The classification of nanomaterials is largely based on their dimensional structures for electron confinement. It includes zero-dimension (0D), one-dimension (1D), two-dimension (2D), and three-dimensions (3D) nanomaterials [11]. Examples of 1D nanomaterials are nanotubes (e.g., carbon nanotubes, CNT), nanorods (e.g., gold nanorods (AuNRs), and silver nanorods (AgNRs)) [12]. The 2D nanomaterials are malleable to form nanosheets (e.g., graphene sheets) and nanoplates (e.g., gold nanoplates). It is ductile and can be drawn into thin nanowires (e.g., gold nanowire and silver nanowires) [13]. Examples of 3D nanomaterials are nanocubes (e.g., gold nanocubes) and nanocages (e.g., gold nanocages). Zero dimensional nanomaterials are also synthesized and some examples are quantum dots and carbon nanoparticles (c-dot) [14]. Based on the morphological crystalline nature, it can be further divided into two types. They are pristine nature and amorphous nature [15]. All of these nanomaterials are used in medical applications. All nanomaterials presented here possess a unique physical and chemical property. A single nanomaterial would enable multifunctional applications in a leading-edge area of biomedical sciences [16]. This is to emphasize that atoms localized in the molecules would have 3D orientations. For example, the molecule methane has tetrahedral structures with 3D orientations. Macromolecules such as DNA and proteins are also present in the nanometer regimes with three dimensions.

## 2. Synthesis of Nanomaterials

The nanomaterials can be synthesized using two prominent approaches. They are top-down and bottom-up approaches. In top-down approaches, the bulk materials are mechanically machined and converted into fine particles in nano dimensions. In bottom-up approaches, the fine particles are assembled to build the nanomaterials through self-assembly or co-precipitation methods [17].

### 2.1. Ball Milling through the Mechanical Method

Ball milling is a mechanical method to fabricate nanomaterials. In this process, the materials are ground in a closed container. Small pebbles made of glass, ceramics, and stainless steel creates shear force during grinding. Bulk materials are subjected to place in the closed container. By grinding process, the bulk materials are converted to fine-tuned nanomaterials [18]. Using this method, we can fabricate metallic hydrides and nitrides. These nitrides possess essential properties that provides a variety of applications. Their hardness and stability are used to cut tools and tool coating in microelectronic applications (e.g., titanium nitride (TiN) alloy). It is synthesized using the reactive ball milling method [19]. In this approach, the bulk metallic powder is placed in the closed container under the purging of the nitrogen gas atmosphere that is subjected to high-energy ball milling. The metallic powders are disintegrated to form tiny particles and oxygen-free active surfaces are created on the nanomaterials [20]. In addition, nanotubes are ground to form very tiny powders. It is created by the collision of two or more pebbles in a closed container that creates very high pressure to get fine nanotubes [21]. The quality of the nanotubes is increased by adding functional moiety on the surface of the nanotube in the container [22]. Some of the factors affecting the dispersion of nanotubes are the size of the pebbles, rotational speed, milling time, and amount of nanotube added [23]. As a result, nanotubes with less than 100 nm can be obtained by the grinding process. Using this process, the carbon nanotubes are transformed into fine carbon nanostructures and during this process, few damages occur on the surface of the nanostructures [24]. The ball milling method is also used in the mechanical alloying method or attrition, a kind of top-down approach. The reduction of particles is done in the griding process by the collision of pebbles that generates a frictional force, which gives rise in pressure, temperature, and internal energy. During this process, the materials are lying in the nanosized regime [25]. The milling speed can be increased through surface functionalization of alkyl or aryl group on the surface of the single-walled carbon nanotubes (SWCNTs), which gives high solubilization in organic solvents [26]. Metastable materials can be produced using high-energy ball milling method by maintaining thermal equilibrium. This process slightly differs from the conventional ball milling methods because the conventional methods cannot produce sufficient energy to mill the materials. For example, nickel-niobium (Ni-Nb) alloy can be produced using high-energy ball milling methods [27]. The advantage of the ball-milling method includes (i) making fine powder; (ii) being suitable to mill toxic materials; (iii) abrasive materials are milled using this technique. The disadvantage of the ball-milling method includes (i) the raise of contamination occurs from wear and tear in balls collision; (ii) increase in machine noise when the concealed cylinder is made of metal and it is reduced when the cylinder is made of rubber; and (iii) it is a time taking process [18].

### 2.2. Physical Vapor Deposition (PVD) Method

Physical vapor deposition (PVD) is a process applied to the synthesis of ultra-thin films and surface coatings. It is used to produce metal vapor that can be deposited on the conductive layer as ultra-thin films and alloy coatings. The whole process is carried in a vacuum held in a vacuum chamber about 10^−6^ torr from a cathodic-arc source. In a clean atmosphere, vacuum deposition is held in the chamber and the metals are deposited as wider or sputtered in the localized area [28]. Reactive PVD is designed in a method to deposit metal on the surface and reactive gas such as oxygen, nitrogen, or methane passed in the vacuum chamber. Plasma, the high energetic beam bombards the metal surfaces ensuring hard and dense coating. Using this method, we can synthesis nano-particles and allow to fabricate nanocomposites [29]. Thin film formation is characterized by the metal ions in the vapor phase obtained from condense phase and return back to condense phase of thin films [30]. The PVD includes evaporation and a sputtering process to fabricate thin films. The procedure for PVD includes sputtering process that is carryover in the vapor phase under supersaturation. In an inert atmosphere the metal vapors are promoted to condense phase, and it is subjected to thermal treatment to get nanocomposites [31]. The advantage of the PVD techniques include (i) having improved properties as compared with the substrate material; (ii) inorganic and few organic materials are used, and it is an ecofriendly approach as compared to electroplating technique [32]. This technique also faces few difficulties such as (i) coating with complex structures; (ii) it is not cost-effective and produces a low output; and (iii) it is a complex process [33].

### 2.3. Lithography

Lithography is a process used in printing that involves a substrate covered with a stamp dip in ink getting the image [34]. The stamp has both hydrophobic and hydrophilic regions where the hydrophobic region takes up the ink while the hydrophilic region will not. Normally, lithography replicates the pattern that resembles the substrate patterning [35]. Two different types of masks are positive and negative which is used to pattern the surface. A positive photoresist is soluble when exposed to light, whereas a negative photoresist is insoluble and makes the photoresist rigid [36]. There are different kinds of lithography used to pattern the surfaces. They are photolithography, UV-lithography, e-beam lithography, soft lithography, scanning probe lithography, and nanolithography [37]. Photolithography is a light-based technique where an image forms on a light projection into the photoresist coated on the substrate (e.g., silicon wafer) [38]. It is widely used technique mainly in the nano-electronics industries to pattern the semiconductors. The specific type of wavelength is used in the UV-lithography in which sub-micron level pattering is possible in the photoresist [39]. Under UV-light exposure, there is a change in the solubility of the solution called developer. After the exposure to the UV light photo-crosslinking occurred on the photoresist and the unreacted pattern was etched away [40]. One of the drawbacks of this technique is the production of free radical during the photo-curable process and DNA damage in photo-initiation processes [41]. E-beam lithography is a technique in which scanning electron beams are used to pattern the surface without a mask and achieve an accuracy of less than 1 nm [42]. The two most common methods for scanning an electron beam are raster scanning and vector scanning. Raster scanning divides an image into pixels, which are printed in a left-to-right or top-to-bottom order, whereas in raster scanning, the beam scans the entire surface, including areas with no features [43].

Soft lithography is a technique that uses an elastomeric stamp to deposit ink on a substrate. It has many advantages over other patterning methods, including a lower cost, simpler setup, and high throughput. It has an accuracy of a wide range from nanometer to micrometer resolution to pattern the substrate [44]. One of the drawbacks of the soft lithographic method is that it requires another lithography method to fabricate the stamp master. If the process is done once and it can be repeatedly used to patter the surfaces [45]. Scanning probe lithography feels the surface to image and modifies the surface with atomic resolution. The probe is used as similar as in atomic force microscopy (AFM) and scanning tunneling microscopy (STM) tips [46]. For example, this technique is used to fabricate graphene nanoribbons by catalyzing graphene oxide in the presence of hydrogen using a platinum-coated AFM tip with a resolution of 20–80 nm [47]. One of the advantages of scanning probe lithography is having the ability to generate features with any geometry and pattern on non-planar surfaces [48]. In addition, this technique is very simple in non-denaturing solution environments and direct interrogation of protein binding events. Integrated circuits with nanopatterning have been synthesized using micro and nanolithography [49]. Lithography is followed by deposition and etching to achieve a high-resolution tomography. Masked lithography and maskless lithography are the two types of lithography. Photolithography is a kind of masked lithography, whereas e-beam lithography is a kind of mask-less lithography [50].

### 2.4. Sol-Gel Method

Sol-gel is a widely used method to prepare nanoparticles. The condensation and hydrolysis reactions are involved in the preparation of nanoparticles. Heat treatment is applied in the intermediate synthesis and is required to ensure the crystallinity of the nanoparticles [51]. The alkoxides serve as a precursor to prepare oxide nanoparticles that interact through molecular forces (e.g., van der Waals forces or H-bonding) and are dispersed in a sol through evaporation or condensation [52]. The precursor of alkoxide is hydrolyzed in the presence of a base or acid, resulting in a polymeric gel. The final product is determined by the rate of condensation and hydrolysis [53]. For example, the smaller size nanoparticles made, the lower the hydrolysis rate. It is an apt process to synthesize composites, oxides, and ceramic nanoparticles with high purity homogeneous distribution [54]. It has an advantage over the conventional oxide fusion method. For example, the xerogel nanocomposites made of iron or silica are produced by the direct mixture of iron III nitrate, using TEOS in a sol-gel formulation. The ferric iron is reduced to metallic iron in the presence of hydrogen gas under a temperature around 400–700 °C. The application of xerogel involves pressing a nanocomposite into a pellet on glass slides for electrical or magnetic conductivity [55]. The advantage of the sol-gel method has high purity and achieves a uniform nanostructure at low temperature in the presence of ligand as a capping agent. The key downside of this method has the high level of impurities from reaction by-products and necessitating posttreatment [56].

### 2.5. Chemical Vapor Deposition Method (CVD)

Chemical vapor deposition (CVD) is a vacuum-based deposition process for producing better-quality, better-performance of solid materials [57]. In this method, thin films deposits over the substrate and involves chemical reactions between species such as organometallic and other gases [58]. The uniqueness of CVD is a multidirectional deposition method to coat over the substrate, while PVD uses a line-of-site impingement method [59]. CVD is commonly used to deposit materials in various forms such as crystalline, amorphous, and epitaxial growth in the microfabrication process [60]. In CVD, a mixture of gases interact chemically over the surface of bulk materials that leads to chemical decomposition forming a dense coating on the base of the material surface [61]. For example, diamond crystals can be deposited over the silicon or molybdenum substrates using the CVD approach. Charged diamond nanoparticles can be produced using the hot filament chemical vapor deposition (HFCVD) process. The diamond nanoparticles are captured using floating and grounded substrates [62]. Some of the substrates are SiO, graphene membranes of copper TEM grid, and carbon. Different types of allotropes diamonds are identified as hexagonal and cubic diamond, i-carbon, and n-diamond particles [63]. The diamond nanoparticles can characterize using Raman spectroscopy that has a sharp peak around 1332 cm^−1^ [64]. The advantages of the CVD process are producing high purity thin films and fabricating abrupt junctions. Disadvantages of the CVD process are the product based on the properties of precursor and poor uniformity [57].

### 2.6. Chemical Co-Precipitation Method

**Figure 1:**
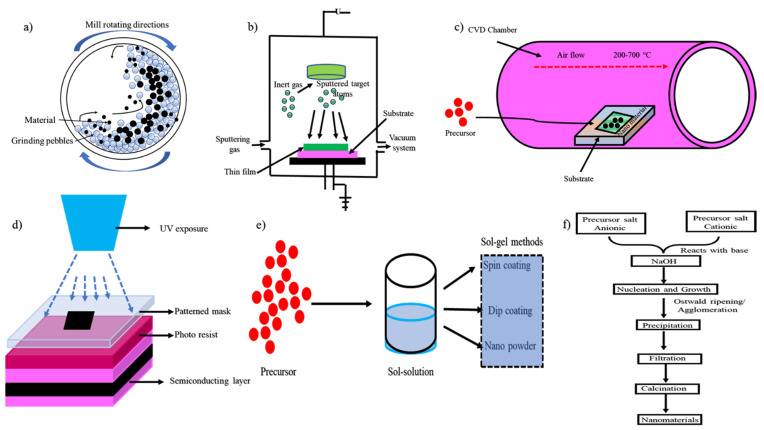
This figure shows a list of top-down and bottom-up approaches to synthesize nanomaterials. (**a**) Ball milling, (**b**) PVD, (**c**) CVD, (**d**) Lithography, (**e**) Sol-Gel method, and (**f**) Co-precipitation method.

The chemical co-precipitation method involves mixing of two different salts that end up with precipitation in an aqueous solvent, especially base [65]. For example, the classical preparation of the magnetic nanoparticles (MNPs) involves through the chemical co-precipitation method. In this process, the Fe^2+^ and Fe^3+^ salts are mixed in the NaOH base [66]. The parameters such as morphology, size, and composition are altered by changing the pH, temperature, ligands, precursor salt, and chemical ratios [67]. The nanoparticles are stabilized by adding appropriate surfactant (e.g., oleic acid), ligand (e.g., aptamer), polymer (e.g., poly(ethylene glycol acrylate)), and inorganic molecules (e.g., NaOH) [68]. Complex metals can synthesize by using this feasible method. For example, the synthesis of iron-chromite obtains by the mixture of iron (III) salt and chromate. In aqueous, the salts from metal ions are formed and then precipitated using ammonium base. The precipitate is decomposed at a high temperature to yield iron (III) chromite [69]. The characteristics of the chemical species synthesized using co-precipitation methods are insoluble and contain smaller particles. The insolubility nature comes from the high supersaturation of the species [70]. The size may vary from smaller to larger. During the nucleation process, larger-sized particles are formed. The factors affecting the formed particles are aggregation and Oswald ripening [71]. The advantages of the co-precipitation methods include simple and direct, control size of the particles based on their compositions, surface of the particles can be modified with various functionalities, requires the low temperature to synthesize particles, avoid the involvement of organic solvent, and is energy efficient [72]. The major disadvantage of this method restricts the involvement of uncharged species, the presence of a trace number of impurities during precipitation, is time-consuming, inhomogeneous in reproducibility, and is affected by the rate of precipitation [73]. Figure 1 describes the list of top-down and bottom-up methods to synthesize nanomaterials.

## 3. Zero Dimensional (0D) Nanomaterials in Theranostics

Quantum dots (QDs) are the emerging nanoparticles used as probes to diagnose and act as labels to identify the tracing of drugs. This kind of nanoparticle can be used for diagnosis and therapy for a disease. QDs probes are made of semiconductor nanoparticles and are luminescent [74]. The excitons are generated when a photon hits the particles whose dimensions are less than the Bohr radius and the energy levels are quantized due to controlled size [75]. The fluorescence comes when the excited electron relaxes and reaches the ground state by releasing the photons with the range of UV to NIR regions. So, the QDs have photoluminescence properties. The examples of quantum dots are CdTe, CdSe, InAs, and InP [76]. A typical quantum dot has a size of less than 5 nm but in medical applications, it can be extended upto 50 nm. The unique properties of the quantum dots are having high photostability and fine-tuning of the optical spectrum [77]. The general physiochemical properties of the 0D materials do not allow electrons to move anywhere in the confined space. Recently, the CdSe quantum dots are prepared using the precursor sodium hydroselenide that reacts with the cadmium chloride in the presence of NaOH base [78]. A soft template is used (e.g., mercaptoundecanoic acid) to form micelles in water. This template gives a confined space that allows the particles in the nanometer regime. This process takes place in the nitrogen atmosphere [79]. This kind of quantum dots can be used in fixed cell imaging, Ex-vivo imaging, in-vivo imaging, and bioanalytics [80].

**Figure 2:**
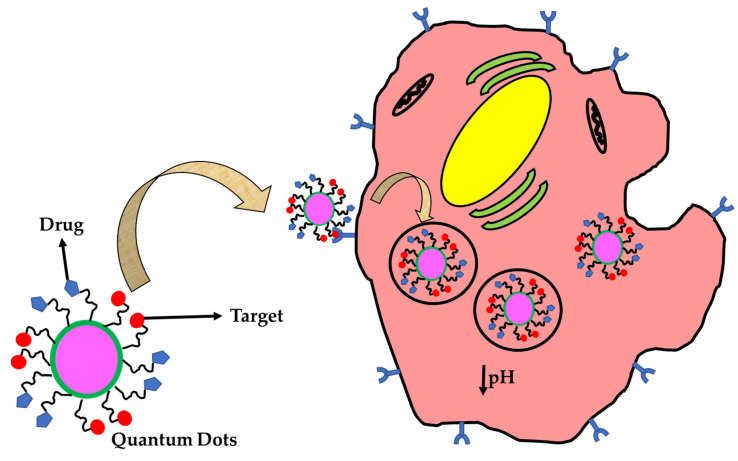
Scheme illustrates the intracellular uptake of QDs.

Hybrid quantum dots are also in the practice to maintain their stability in the biological fluids (e.g., protein-QDs nanohybrids) [81]. Gelatin-QDs show no toxic effects on cells up to 5 mg/mL [82]. A multifluorescent nano-hybridis prepared using the CdTe/CdS nanoparticles [83]. Zein-QD nanohybrids can employ for drug delivery applications for the delivery of 5-fluorouracil [84]. The biocompatibilityhave tested using MCF-cell lines and showed more than 80% cell viability. The hybrid QD can synthesize using polysaccharides, lipids, and polymers [85]. NIR and IR QDs are synthesized for bioanalytics applications. The species used to synthesize QDs are Cd (II) and Pb (II) by convention and recently they can synthesize using Ag (I) and Cu (I) species [86]. These QDs generate fluorescent absorbance in the range between 650 to 800 nm in the NIR region and are greater than 800 to 1300 nm in the IR region where the biological tissue obtains low absorbance [87]. Carbon-based quantum dots are employed in theranostics applications. The carbon quantum dots are also known as C-dots, which are derived from carbonaceous material with a size less than 10 nm [88]. It has many optical properties and includes unique properties such as biocompatibility and high photostability. Originally it is found as a byproduct during the synthesis of carbon nanotubes. It is the better alternative for metal-based fluorescent nanomaterials [89]. There are no such difference between QDs and C-dots, rather, QDs are metal-based whereas C-dots are derived from carbon-based materials. A minute difference may occur based on fine-tuning and its photoluminescence bandwidth. The emission comes from the C-dots and depends on laser excitation, whereas QDs exhibit based on their size [90]. Recently, the C-dots are used to visualize the cell components such as cell membrane, cytoplasm, endoplasmic reticulum, Lysosomes, Mitochondria, Golgi bodies, and Nucleus [91]. Recently, doxorubicin can load in the composites of C-dots (e.g., arginine-glycine-aspartate-C dots), which apply for drug delivery and targeted imaging in therapy [92]. Table 1 shows the list of 0D nanomaterials with specifications and applications. The major therapeutic application of 0D materials has a high chance of renal clearance as compared to other dimensional nanomaterials. So, it can be used to deliver drugs at the target sites and the materials are removed through the excretory track and make sure that the materials are bio-compatible. Figure 2 describes the intracellular uptake of QDs for theranostics.

## 4. One Dimensional (1D) Nanomaterial in Theranosis

Nanorods and nanotubes are included in the 1D nanoparticles, which are predominantly applied in theranostics [97]. The physiochemical properties of the 1D nanomaterials can confine electrons in two directions. Nanorods can synthesize by various methods such as polyol and surfactant-assisted methods [98]. In the soft template-assisted method, seed solution has been prepared and added to the growth solution. CTAB and CTAC are the surfactant involved in the synthesis of nanorods [99]. Recently, the phospholipids functionalized gold nanorods are used for in-vivo imaging applications. Here the CTAB is replaced with other series of phospholipids such as DOPC (Di-palmitoyl phosphatidylcholine) and DSPE (Di-stearoyl phosphoryl ethanolamine) [100]. The surfactant displacement can achieve through sonication and purification using centrifugation. The CTAB removal can characterize using NMR spectroscopy, SERS, and zeta potentials [101]. The stability of the nanorods is analyzed using different biological media such as blood plasma, serum, cell culture media, and different pH and buffers [102]. These nanoparticles hold their stability without aggregation in biological fluids. The biocompatibility is tested in murine models in-vivo and has high photothermal efficiency when it is irradiated with NIR lights [103]. The heat generated by nanorods is sufficient to kill the cancerous cells and opens a new avenue in cancer nanomedicine [104]. The working principle of gold nanorods is the oscillation of the outermost valence electron resonant at a particular frequency when it experiences the photon hitting, leading to generating heat. It is believed that the generated heat destroys the cancerous tissues. In addition, new nanorods have been synthesized using bismuth sulfide iodide (Bi_19_S_27_I_3_) for photothermal therapy, which covers absorption from visible to NIR and IR regions. It has a photothermal efficiency of about 42% and is biocompatible, which exhibits a lethal effect on cancerous cells. Solvothermal synthesis are employed to fabricate bismuth sulfide iodide nanorods [105].

Nanotubes are another nanomaterials used for theranostics applications. The common methods used to synthesize nanotubes are CVD, laser ablation, arc discharge [106]. Among these, CVD is the most robust method due to being cost-effective, controllable, and having high throughput. There are many techniques to fabricate carbon nanotubes. The nanomaterials are controlled with different parameters such as maintaining the condition of low pressure in an inter atmosphere using argon gas, having a high yield of up to 60–90%, and maintaining the temperature of 500–1200 °C [107]. Single-walled and multiwalled carbon nanotubes have been fabricated with a diameter of 0.6 to 4 nm and 10–240 nm, respectively. Single-walled carbon nanotubes (SWCNTs) can be used as theranostics agents due to their high photothermal ability and NIR absorptivity. It is considered a better alternate agent as compared to conventional radio or chemotherapeutics [108]. One of the main advantages of SWCNTs shows the destruction of the cancerous cells during aggregates and are photo-acoustically inactive when they are scattered. It explains that the cancerous cells die in SWCNTs aggregation, not in the isolated uptake. So that the normal tissues cannot be injured during the photothermal process (Table 2) [109].

**Figure 3:**
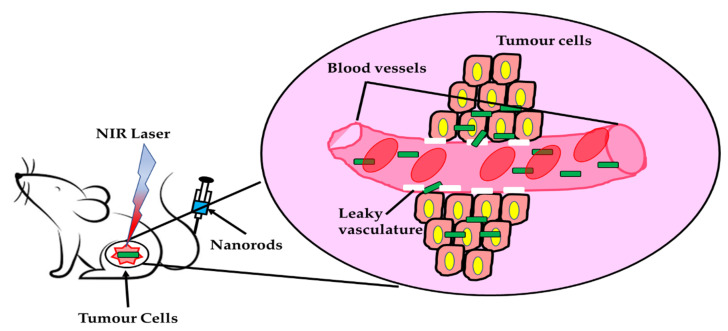
Represents the targeting of nanorods for theranostics.

Another nanotubes synthesized from different materials are also used in theranostics applications. For example, hybrid nanotubes (HNTs) are developed using porphyrin-covered silica nanomaterials using electrostatic interactions and π-π stacking through the sol-gel process. It is used to track macrophages in-vivo [110]. Also, magnetic nanotubes are employed in theranostics. The magnetic nanotubes are synthesized using the hydrothermal method in which the iron chloride is mixed with sodium salts and the reduction occurred upon heating. Then, the synthesized iron oxide is stabilized using PVP [111]. The magnetic nanotubes are applied for hyperthermia in cancer theranostics [112]. The therapeutic applications of the 1D can be used for passive targeting in cancer therapy. Nanowires will allow materials for prolonged circulation until it reaches the target. Figure 3 displays the targeting of nanorods to the cancerous cells for theranostics.

## 5. Two Dimensional (2D) Nanomaterials in Theranostics

Nanosheets and nanoplates are employed as 2D nanomaterials in theranostics applications [118]. The 2D nanomaterials can confine electrons in only one direction. For example, graphene is the allotropic structure of carbon that exhibits in the two-dimensional network. An increase in surface area is observed when there is decrease in the stacked layer of the graphene sheet. Based on the arrangement, it could be a single-layer or multi-layered framework [119]. The physiochemical properties of the graphene sheets are sp^2^ hybridized, high Young’s modulus, thermal conductivity, and electrical conductivity [120]. The graphene can be synthesized by both top-down (e.g., exfoliation method) and bottom-up (e.g., self-assembly or CVD) methods. It also enhances biological properties such as cellular uptake, interacting with cells, transport across the blood-brain barrier (BBB), and renal clearance [121]. Advancement in the synthesis of multifunctional nanoparticles can be employed as diagnostic and therapeutic effects in monitoring the diseases [122]. Manipulating the surfaces, the graphene are reacting with an acid to get more reactive oxygen and hydrophobic interaction will be applied to encapsulate the high loading capacity of the drugs. These nanosheets can be employed in multi-drug resisting cancer therapy [123].Graphene oxide (GO) and reduced graphene oxide (rGO) are also employed in biological applications, especially in theranostics. The main difference between GO and rGO is based on the presence of surface oxygen and its conductivity. The rGO shows high conductivity and is used as electrode material for supercapacitor applications [124]. Recently the PEGylated-GO is used to load the tumor-targeting peptide and anti-cancer drugs for targeted cancer therapy. Doxorubicin (DOX) is chosen as an anticancer drug and is delivered to the cells due to its π–π interaction and weak hydrogen bonding between DOX and GO [125]. The functional rGO is employed to load genes in higher-order efficacy for gene therapy. It is an excellent photo-absorber and has absorption at the NIR region. It shows high photothermal as compared to GO-based nanomaterials [126]. Other nanomaterials such as MXenes and gold nanoplates are also employed in theranostics applications (Table 3) [127]. Therapeutic applications of 2D nanomaterials can have high photothermal efficiency, which can’t be given by other-dimensional nanomaterials.

## 6. Three Dimensional (3D) Nanomaterials in Theranostics

Nanocubes and nanostars are employed as 3D nanostructured materials in theranostics applications [133]. The physicochemical properties of 3D nanomaterials cannot confine electrons in any direction. For example, silver nanocubes and gold nanorods have been employed in photothermal applications. Here, silver nanocubes are synthesized using the polyol method with the assist of sulfur-mediated reduction process in the presence of PVP. These nanocubes are considered as templated to synthesis gold nanocage and involved in photothermal applications [134]. The dissolution of silver nanocubes are performed using chloroacetic acid (CAA) and the displacement of gold atoms have done through galvanic replacements [135]. The gold nanocage utilizes the principle of surface plasmon resonance (SPR). In this principle, a laser hits the outer most electron of the nanoparticle and gets oscillated and absorbs the NIR lights [136]. The heat generated by the nanoparticles is enough to kill the cancerous cells and so it is applied in the theranosis. Cobalt-iron oxide (Co_x_Fe_3_-xO_4_) nanocubes are employed in theranosis applications. The magnetic nanocubes are synthesized using a thermal decomposition process in which cobalt, iron, decanoic acid are mixed in squalene and benzyl ether. Now, the solution is treated with an increase in temperature from 65 to 305 °C by initially applying pressure [137]. The black-colored solution is purified with acetone: IPA mixture under centrifugation. Finally, the mixture are stored in the chloroform for further studies. The hydrophobic iron oxide nanocubes have phase- transferability by modifying the nanocrystal surface with PMAO (Poly(maleic anhydride-alt-1-octadecene)) polymer. Finally, the solution is stored in borate buffer at alkaline pH (pH = 9) for better solubility [138]. The high coercivity and magnetization property of core/shell nanocubes is used for magnetic hyperthermia. It is well known that magnetic hyperthermia is used in cancer therapy where the electromagnetic energy is converted in the form of heat, and this is due to magnetic hysteresis of the nanomaterials [139]. The therapeutic applications of 3D nanomaterials is having more space to accommodate enormous amount of drugs and produce high heat in hyperthermia, which cannot be given by other-dimensional nanomaterials.

**Figure 4:**
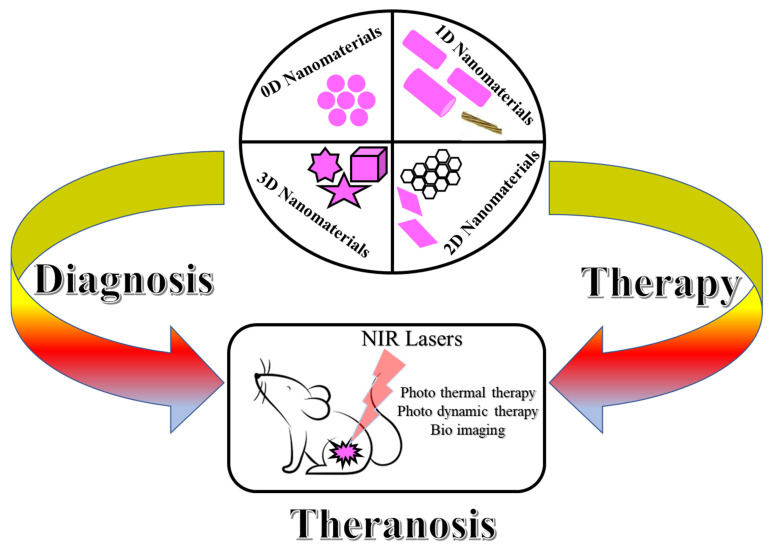
The scheme represents the application of 0D, 1D, 2D & 3D nanomaterials application in theranosis.

Deep tissue attenuation is a challenging treatment in cancer theranostics. Recently, Naresh Kuthala et al., developed lanthanum hexabromide nanocubes (LaB_6_-NCs), which covers a broad range of biological window (NIR I & II- window) with a higher-order magnitude of five, over the conventional dyes and organic photo-sensitizers. It acts as a bimodal theranostics agent for T_2_-weighted MRI and CT modalities. The nanomaterials-mediated PDT is used to conquer the problem of hypoxia using intracellular water as a free radical generating source to treat the tumor. Up-taking of the intracellular oxygen during PDT is worsening the effect of the hypoxia tumor. So, the developed LaB_6_-NCs can locate the presence of a tumor and helps to remove it [140]. In Figure 4, it is represented that different dimensional nanomaterials can be applied in diagnosis and therapy for the application of theranostics in the field of biomedical sciences.

### Characteristics of Theranostic Nanomaterials

The concept of theranostic nanomaterials has been discussed in the previous reports [79,80,81,82,83,84,85,86,87,88,89,90,91,92,93,94,95,96,97,98,99,100,101,102,103,104,105,106,107,108,109,110,111,112,113,114,115,116,117,118,119,120,121,122,123,124,125,126,127,128,129,130,131,132,133,134,135]. According to Ferrari’s classification [141], theranostic nanomaterials are categorized into three components based on their role and physical locations: (a) biomedical payload: includes imaging agents such as organic dyes, MRI contrast agents, CT contrast agents, etc. and therapeutic agents such as anti-cancer drugs, DNA, proteins, hyperthermia-inducing nanoparticles, etc.; (b) carrier: includes providing physical protection for the biological payload during delivery to the specific target site under physiological conditions. In particular, organic-based carriers such as dendrimers, polymers, lipids, and inorganic-based carriers have been developed; (c) surface modifier: is a component attached to the surface of the carrier to provide additional properties for the theranostic nanomaterials such as long duration time, ability in barrier-penetration, and target specific binding.

## 7. The Recent Process on the Nanomaterials in Theranosis

Conventionally, the QDs are synthesized using heavy metals such as cadmium and selenide materials. Recently, the QDs are developed using CdSe/ZnS core-shell nanomaterials. The core-shell nanomaterials are developed to avoid heavy metal-induced toxicity. The CdSe acts as a core and the ZnS acts as a shell, which acts as the metallic biocompatible protective outer coat. It has an average size of less than 10 nm and has a strong absorption at 579 nm. The Cd109 acts as a SPECT imaging agent incorporated for imaging and diagnostic purpose to identify the tumors [93]. In another work, the authors used CdTe and CdS QDs are functionalized with BSA proteins to reduce the heavy metal-induced toxicity. It has an average size of 550 nm and has fluorescence with excitation at 400 nm and the emission range from 528 to 650 nm. The QD-BSA is used for the long-term fluorescence and the emission decreased by 4% when it is irradiated at 365 nm laser for 1 h [84]. The carbon QDs are arrived to avoid the usage of heavy metal and to reduce toxicity. Recently, amino acids, quinones, and citric acids are used to synthesize the carbon QDs, which are having an average size of 3 nm and have strong absorption at three different ranges at 230, 280, and 650 nm. It is used for Lat-1-mediated targeting tumor theranostics [94]. These recent processes on 0D nanomaterials are summarized in Table 1 with specifications and applications.

Nanorods and nanowires are examples of 1D nanomaterials. Conventionally, the nanorods are synthesized by conventional heating or microwave irradiation methods. Recently, the template-assisted methods can yield more nanomaterials. The soft template-assisted synthesis is more attractive and the manipulation happens at the molecular self-assembly level. Recently, the gold nanorods with the size range of 2.9–4.2 aspect ratio has absorption at 770–811 nm. The petawatt lasers are used to treat lung cancers instead of conventional lasers and the energy of the laser is about 19 mJ/cm^2^ [113]. The CTAB-assisted synthesis of gold nanorods (AuNRs) shows cation-induced toxicity. Avoiding the use of such toxic effects, the gold nanorods are functionalized with silica, the inert materials to reduce the toxic effect on cells [116]. Lasers are used to trigger the AuNRs to produce heat which will act as stimuli for killing the cancer cells. In recent advancement, Gadolinium (Gd) are tagged with AuNRs for MRI theranostics and imaging [117]. Nanowires are another theranostic agent used to treat cancer using NIR irradiations [115]. These recent processes on 1D nanomaterials are summarized in Table 2 with specifications and applications.

Nanosheets and nanoplates are an example of 2D nanomaterials. Graphene and graphene oxide nanomaterials are conventional nanomaterials having toxicity on cells. So, the advancement of the functionalization technique coats PEG on the surface of graphene/GO/rGO in the treatment of cancer [131]. Recently, MXenes sheets are considered as converting the materials from the state of light to heat with 100% photo-thermal efficiency [128]. The advancement of scientific methods develops boron nitride nanosheets which cover poly-phenolics such as tannic acid to reduce toxicity and are used as phototherapy guided by MRI [129]. These recent processes on 2D nanomaterials are summarized in Table 3 with specifications and applications.

## 8. Theranosis: Future Direction and Challenges

The major challenge faced by the scientific community in cancer therapeutics is intra-operative cancer cells for the accurate detection and early diagnosis for the treatments. The FDA approves a limited number of organic dyes due to photobleaching capacity and toxicity. It does not have long-standing storage to diagnose the disease. Quantum dot is the better alternative for conventional dyes and has the fine-tuning ability for diagnosis. Differentiating normal tissues from cancerous cells is one of the challenges facing biomedical sciences. These nanoparticles have multifunctional targeting ability to locate the target site and can have the ability to deliver drugs for therapy. After reaching the target, nanomaterials are used to treat the tumour cells using phototherapy. Heterogeneity is another challenge faced by the scientific community. This multifunctional targeting can give a solution in cancer theranostics. Developing nanoprobes with NIR absorption is emerging where the biological tissues exhibit low absorption and are easy for early diagnosis. The development of light to heat converting probes are needed for theranosis.

## 9. Conclusions

Theranostics is an emerging field in nanomedicine in which nanoparticles are employed as a diagnostic and therapeutic agent to cure diseases. Conventionally, in theranostics, the organic and inorganic materials are employed by lacking the multifunctional ability to treat the diseases. Conventional treatments perform low inefficiency to cure the diseases. In this queue, nanotechnology is an emergent field employed with different dimensions of the nanoparticles having unique properties to treat the diseases. For the zero-dimensional nanoparticle particularly, the quantum dots make an alternative to the conventional dyes to avoid the photobleaching ability and prolonged fine-tuned emissions for tracking the cells or molecules in the living systems. Since the quantum dots exhibit heavy metals that induce cytotoxicity, the carbon-quantum dots are the better alternative to avoid heavy metal-induced cytotoxicity. Controlling the size in any of the dimensions is a challenging issue in carbon quantum dot-based nanomaterials. For an instant, the microwave-assisted one-step polyol method is used to synthesize green fluorescent carbon dots and used as nanoprobes in theranostics. Gadolinium functionalized fullerenes (Buckyballs) are employed in MRI theranostics.

Synthesis of NIR-absorbed nanomaterials has emerged in biological applications since the biological tissues exhibit low absorbance in that region. The one-dimensional nanomaterials, gold nanorods (AuNRs) are the better alternative for other organics in imaging and photothermal therapy. When the light hits the AuNRs, the outermost electrons get oscillated, which exhibit the surface plasmon resonance, which is resonant at a particular frequency and has maximum absorbance in the NIR region. The oscillated electron induces heat and the heat is enough to kill the cancerous tissues. CNTs are another 1D nanomaterials that can be employed in theranostics applications. CNTs can be used as NIR absorbance in photothermal therapy. It has a long tube used to encapsulate a large number of drugs with a very high efficiency. For an instant, the encapsulation efficiency of PEG-FA CNT is about 149.3%, whereas MWNT-HCPT shows 16% drug loading efficiency. The difference in drug loading is observed due to increased surface area obtained in SWCNTs.

The 2D nanomaterial includes graphene nanosheets employed as a theranostics agent in nanomedicine. Recently, the chlorambucil drug-loaded gelatin-coated folate functionalized rGO shows the encapsulation efficiency of about 56% from the wt. of 10 mg/mL. In recent years, the nanocomposites made of rGO-Fe_3_O_4_ exhibit the superparamagnetic property and have the capacity to increase the surrounding temperature of ~20 °C in concise with the initial temperature. The photothermal therapy reduces the cancerous cell viability to 23.7% for 100 µg/mL of nanomaterials. Another 2D nanomaterial is MXenes, recently the researchers identify that Nb_2_C exhibits photoabsorption at the second-biological window, i.e., NIR-II from 1000 to 1350 nm with the highest photoconversion efficiency of about 50%. The temperature of Ti_2_C_3_-SP nanosheet is made fast to approach up to 55 °C after 6 min exposure to NIR laser irradiation, having high photothermal conversion.

The 3D nanomaterials include nano-cubes and are employed as theranostics agents in biological applications. The gold nanocubes have high photoluminescence (PL) that shows 200 times greater than AuNRs for cancer cell imaging about 4 × 10^−2^ PL and is suitable to facilitate photothermal effects on cancer cells. These potential nanomaterials have a wide range of applications and have a bigger effect on theranostics in nanomedicine.

## Figures and Tables

**Table 1:** Summarizes the list of zero-dimensional (0D) nanomaterials (e.g., quantum dots), their specification, and applications.

S. No.	Nanomaterial	Elements	Size (nm)	Absorption (nm)	Functionalization	Application	Ref.
1	Quantum dots	CdSe, ZnS	<10	579 nm	Core/Shell-ZnS	Cd109-SPECT imaging agent incorporated for imaging and diagnostics to identify the tumour	[93]
2	Quantum dots	CdTe, CdS, BSA	~550	λ_ex_ = 400 nm λ_em_ = 528 to 650 nm	Protein (BSA)	QD-BSA was used for long term fluorescence observation because emission decreased by 4.06% after being irradiated at 365 nm for 1 h	[84]
3	Carbon quantum dots	large amino acid-mimicking (LAAM), tetramino-anthraquinone (TAAQ), and citric acid	~3	230 nm, 280 nm, 650 nm	α-carboxyl, amino groups	LAT-1-mediated targeting tumour theranostics	[94]
4	Quantum dot	Mn, ZnS	218	287	Chitosan biopolymer and conjugated with folic acid	Diagnosis and treatment of anticancer activity of 5-fluorouracil for breast cancer therapy	[95]
5	Quantum dot	In, P, Zn	15–20	645	Mercapto-succinic acid	This is a non-cadmium based QD used in diagnostic imaging in the early detection of cancer	[96]

**Table 2:** Summarizes the list of 1D nanomaterials employed in theranostics applications.

S. No.	Nanomaterial	Elements	Size (nm)	Absorption (nm)	Functionalization	Application	Ref.
1	Nanorods	Au	2.9–4.2 (aspect ratio)	770–811 nm	Citrate, CTAB	Petawatts (PW) lasers are used to treat lung cancer cells instead of continuous wave (CW) lasers in theranostics. The laser energy used was 19 mJ/cm^2^.	[113]
2.	Nanowire	Magnetic polypyrrole, PEI, Antibody	10	---	Biotin, Antibody	Magnetic nano wires are used to recover rare circulating biomarkers which improves cancer diagnostics and prognostics	[114]
3	Nanowire	Au, Si	Au~500 nm, Si nanowire~17 aspect ratio	530 nm	Gold nanoparticles decorated on silicon nanowire	AuNP@SiNW acts as a NIR hyperthermia agent which destroys the cancer cells within 3 min upon NIR radiations	[115]
4.	Nanorods	Au, Si	3–4 aspect ratio	AuNRs~600 nm Au@SiNRs~725 nm Au-Janus~650 nm	AuNRs functionalized with silica nanoparticles	Au@Si-Janus nanoparticles act as a carrier to deliver imaging agents and drugs. It also useful for combined photo-thermo or chemo cancer therapy	[116]
5	Nanorods	Gd, Au	20–40	>750 nm	PEG functionalized Gd@AuNRs	Gd@AuNRs acts as a strong theranostics agent to image and treat MIAPaCa-2 cells. For NIR 808 lasers used and for MRI T_1_ features at 7T	[117]

**Table 3:** Summarizes the list of 2D nanomaterials employed in theranostic applications.

S. No.	Nanomaterial	Elements	Size (nm)	Absorption (nm)	Functionalization	Application	Ref.
1	MXene	Ti_3_C_2_	500	800	---	MXene acts as light to heat convert material with 100% efficiency in PTT	[128]
2	Nanosheet	Boron nitride	100	650	Tannic acid (TA)	The TA-Fe coordinated complex on boron nitride nanosheet configuring T_1_ weighted MRI- Theranostics. It is also useful for MRI guided photo-therapy	[129]
3	Nanosheet	Graphdiyne (GDY)	360	700	PEG	GDY-PEG acts as a photothermal-acoustic wave transducer in PAI and PTT for treating cancer	[130]
4	Nanosheet	Graphene	50	---	PEG, BPEI, DOX	The graphene oxide nanocomposites act as theranostics agents for UCL image-driven combinatorial PTT and chemotherapy to treat cancer. The NIR laser used at 980 nm with 13.5 photothermal conversion efficiency	[131]
5	Nanosheet	(Gd^3+^)MoSe_2_	100–150	700–850	PEG	Gd provides T_1_ weighted MR-imaging. (Gd^3+^)MoSe_2_ acts as photothermal agents in cancer therapy	[132]

## Data Availability

Not applicable.
